# Correlation and Conversion of the Normalized Constant Score and Patient-reported Outcomes in Shoulder Arthroplasty

**DOI:** 10.5435/JAAOSGlobal-D-22-00247

**Published:** 2023-01-04

**Authors:** Joseph J. King, Jorge N. Gil, Thomas W. Wright, Terrie Vasilopoulos, Aimee M. Struk, Ashlie Hoover, Brendan A. Williams, Kevin W. Farmer, Bradley S. Schoch

**Affiliations:** From the Department of Orthopaedic Surgery and Sports Medicine, University of Florida, Gainesville, FL (Dr. King, Dr. Gil, Dr. Wright, Dr. Vasilopoulos, Ms. Struk, Ms. Hoover, and Dr. Farmer); the Department of Orthopaedic Surgery, Childrens Hospital of Pennsylvania, Philadelphia, PA (Dr. Williams); the Department of Orthopaedic Surgery, Mayo Clinic, Jacksonville, FL, (Dr. Schoch); and the Department of Anesthesiology, University of Florida Gainesville, FL (Dr. Vasilopoulos).

## Abstract

There is a paucity of literature describing correlations between the Constant score and commonly used patient-reported outcome measures (PROMs) in shoulder arthroplasty. This study aims to establish the correlation of and conversion between the normalized Constant score (nCS) to commonly used patient-reported outcomes in shoulder arthroplasty patients.

**Methods:**

A retrospective review of a prospectively collected research database was done from 2003 to 2014. Inclusion criteria were primary shoulder arthroplasty (anatomic or reverse) and minimum 2-year follow-up. Preoperative and postoperative outcomes scores (1-year and 2-year) were prospectively collected and included the nCS, Simple Shoulder Test (SST), Shoulder Pain and Disability Index (SPADI), and American Shoulder and Elbow Surgeons (ASES) Score. The nCS was correlated with PROMs (Pearson correlation for SPADI/ASES scores and Spearman correlation for SST).

**Results:**

A total of 762 shoulders in 721 patients with 1661 individual clinical encounters were included. The average age of included patients was 67.7 years, 48% of patients being female. Reverse total shoulder arthroplasty (RSA) was more commonly done compared with anatomic total shoulder (aTSA) (57.3% vs. 42.7%, respectively). The nCS correlated strongly with the PROs overall: ASES (0.893, *P* < 0.001), SPADI (−0.896, *P* < 0.001), and SST (0.873, *P* < 0.001). Correlations were similar overall between aTSA and RSA. Preoperative correlations between the nCS and the PROMs were on the high side of moderate correlation with RSA (*R* = 0.621 to 0.659) and on the low side of a strong correlation with aTSA (*R* = 0.704 to 0.705) except for the SST (*R* = 0.608). The 1- and 2-year postoperative time points all showed strong correlation of the nCS with PROMs, except the SST in RSA (*R* = 0.694).

**Conclusion:**

The nCS shows high correlation with ASES score, SPADI, and SST in shoulder arthroplasty patients. This suggests that PROMs may be able to be used for shoulder function assessment without the need for physician input for large cohort studies. In addition, the conversion equations generated here may provide utility in the evaluation of shoulder arthroplasty outcomes across studies in systematic reviews and meta-analyses.

The use of shoulder arthroplasty for the treatment of end-stage arthritis and irreparable rotator cuff tears has increased dramatically over the last few decades.^1^ Outcomes of shoulder arthroplasty are generally good,^2,3^ with success being demonstrated across diverse patient cohorts. Recent work has focused on optimization of patient outcomes across a spectrum of diagnoses. There has been a general shift away from clinician-measured outcomes and toward patient-reported outcomes as a way of evaluating surgical success after shoulder surgery from the patient's perspective. The use of subjective measures gives surgeons a better understanding of how the patients perceive their outcomes, and this has become important in the current healthcare environment, given the focus on cost-effectiveness and healthcare utilization.

Numerous patient-reported outcome measures (PROMs) are used to assess shoulder function.^4,5,6,7^ There remains no single commonly used score, nor consensus as to which scores are best, to assess shoulder arthroplasty outcomes. Often, scores are obtained based on surgeon preference and may be based on training, institutional licenses, or concern about patient survey burden. This has led to a heterogenous utilization of PROMs across the literature. In an effort to facilitate cross-study outcome comparison, several studies have evaluated the correlation between certain shoulder PROMs.^4,8,9,10,11^ Yet, there remains a paucity of shoulder arthroplasty literature comparing PROMs with outcome measures requiring clinician input, such as the Constant-Murley score.

The Constant score is a combination of both patient-reported and clinician-measured variables, including pain, function, strength, and range of motion (ROM),^12^ and is commonly used throughout the shoulder literature.^5,13^ Unlike PROMs, the Constant score requires clinician input of objective measurements, including ROM and strength measured. The normalized Constant score (nCS)^14^ is a variation of the Constant score that includes age and sex for the calculation, which has been shown to be important in evaluating older patients due to the normal aging process of losing overhead ROM and strength.^15^

## Rationale

The Constant score is commonly used for shoulder surgery outcomes, but the addition of physician input is a weakness given the variations in the way strength and ROM are measured.^13,16^ More knowledge about how the Constant score correlates with PROMs is important and may have implications for improved meta-analyses and systematic reviews given the extensive use of this score in Europe.^4,5^ Questionnaire burden and fatigue can also be an issue for patients, so a better understanding of the correlation between outcome scores can help surgeons limit the number of questionnaires that patients undergoing shoulder arthroplasty need to complete.

The primary aim of this study was to assess the correlation between the nCS and commonly used PROMs requiring no clinician input. We hypothesized a strong correlation would exist between the Constant score and the PROMs used at our institution. We secondarily sought to develop a conversion algorithm between the Constant score and the PROMs assessed.

## Patients/Methods

### Study Design and Setting

A retrospective review was done of a single institution's prospectively collected shoulder arthroplasty database from February 2003 to December 2014 after receiving Institutional Review Board approval. The analyzed database included patients undergoing shoulder arthroplasty by four fellowship-trained orthopaedic shoulder surgeons (K.W.F., J.J.K., B.S.S., and T.W.W.).

The study database includes multiple patient and physician-derived functional outcome scores at standardized time points (preoperatively and 3, 6, 12, and 24 months postoperatively) throughout the preoperative and postoperative period for all patients undergoing shoulder arthroplasty. For the purposes of this study, one clinician-derived score (the nCS) and three PROMs measures (American Shoulder and Elbow Surgeons [ASES] Score, Shoulder Pain and Disability Index [SPADI], and Simple Shoulder Test [SST]) were evaluated from the database. Patient questionnaires are completed at the time of follow-up clinic appointments. Clinician measures for the nCS including active and passive range of ROM measurements were done in a standardized fashion by our research team at each visit using a goniometer, while strength measurements were obtained using a Lafayette Hand-Held Dynamometer (Lafayette Instrument Company).

For the purposes of this study, only the preoperative, and 12- and 24-month postoperative scores were evaluated. The other database time points collected were not evaluated because of the inferior correlation strength identified between PROMs at earlier time points,^17^ and the fact that cross-study outcome comparisons focus predominantly on late-stage patient outcomes scores.

### Participants

Identification of eligible subjects was done through a query of the shoulder arthroplasty database. Study inclusion criteria were adult patients undergoing primary anatomic total shoulder (aTSA) or reverse total shoulder arthroplasty (RSA) with completion of the nCS, ASES score, SPADI, and SST at given clinical time points. Patients undergoing revision surgery and patient's time points without completed questionnaires and necessary clinician provided data were excluded. Given that each patient acted as their own control, we included all diagnoses and did not exclude patients if they had complications as long as they had all required outcome measures at the specified time points.

### Surgical Procedure and Rehabilitation

One of four fellowship trained shoulder surgeons conducted all of the shoulder arthroplasties. All procedures were done under general anesthesia commonly with regional anesthesia augmentation with the patient in a lazy beach chair position. A deltopectoral approach was used in all cases. The decision for total shoulder arthroplasty versus RSA was made based on preoperative imaging along with the clinical appearance of the rotator cuff per the operating surgeon's judgement. Shoulder arthroplasty was done in the standard fashion. A similar rehabilitation protocol was used for all patients and consisted of a home exercise program under the direction of an occupational therapist at postoperative clinical visits. Shoulder ROM was restricted for 3 weeks. External rotation was limited to neutral, and internal rotation was limited to the abdomen for 6 weeks postoperatively. A sling was used at all times during the first 6 weeks except during the home exercises and for hygiene purposes. Strengthening was started 12 weeks postoperatively.

### Outcome Scores

The Constant and Murley^18^ score consists of four main features, two subjective and two objective, related to shoulder pathology. Pain, activities of daily living (ADL), ROM, and strength are all assessed. Pain and ADL sections can receive up to 35 points, and ROM and strength can receive 65 points. Subjective findings are answered by the patient, whereas ROM and strength are reported by the examiner. Normalization of the Constant score was designed to account for how age and sex affect the ROM and strength component of the score,^14^ therefore allowing for a more accurate and valid representation of function based on age and sex.^14^ In this study, the nCS was chosen for correlation and conversion given the wide range of ages for shoulder arthroplasty patients and inclusion of much older patients, which has shown to have a notable effect on the Constant score in healthy patients.^14^

The ASES score is calculated using the patient self-evaluation section of the ASES assessment form. The patient self-evaluation section contains pain and instability scales and an ADL index.^19^ The pain and instability scales and the activities index are weighted equally for a combined score of 100, with zero being the worst score and 100 being the best score.

The SPADI is a patient questionnaire developed to measure shoulder pain and disability.^20^ The questionnaire contains 13 items, 5 that evaluate shoulder pain and 8 that assess shoulder disability. Responses are summed and transformed into a combined score of a 100, with zero being the least shoulder impairment and pain and 100 being the worst shoulder impairment and pain.

The SST is a patient questionnaire focusing on the shoulder function of patients with shoulder pathology conditions.^21^ It consists of 12 binary “yes/no” questions about whether patients can perform ADLs that can be done by people with normally functioning shoulders. Responses are summed out of a maximum score of 12, with zero being the worst score and 12 being the best score.

Correlations were calculated between the nCS and the PROMs (ASES score, SPADI, and SST) for each time point and for each arthroplasty type. These correlations were then used to form conversion equations between the nCS and the PROMs.

### Statistical Methods

Descriptive statistics were done to analyze cohort demographics. For each outcome score, an average score with standard deviation was calculated overall, at every included clinical time point, and for each procedure type (TSA and RSA). Scatter plots were created for each pairwise outcome score comparison overall. Correlative strength for nCS versus ASES and SPADI score comparisons was assessed using a Pearson correlation coefficient. The Simple Shoulder Test was treated as an ordinal variable requiring the use of a Spearman correlation coefficient to assess correlative strength with the Constant score. Correlation coefficients of 0.4 to 0.7 were considered moderate, while scores greater than 0.7 were considered strong. Conversion equations between the nCS and each other PROM (ASES, SPADI, and SST) were calculated using the best-fit line for each comparison (linear model). Each equation was developed in 75% of the sample and subsequently tested in the remaining 25% (internal validation). Testing and internal validation samples were selected through stratified randomization to ensure that development and testing samples had similar characteristics (age, sex, implant, and time of measurement). In the testing sample (25%), mean differences with 95% confidence intervals were calculated between actual values for each outcome score and their corresponding predicted values from conversion equations. Pearson correlations between actual and predicted values were also calculated. In addition, within-individual differences between actual and predicted values were computed. *P* < 0.05 was considered statistically significant for inferential tests. Statistical analyses were done using JMP Pro 14.0 (SAS Institute) and SPSS software (version 25.0; IBM).

## Results

Complete data sets meeting inclusion criteria were available for 762 shoulders in 721 patients with a total of 1661 individual clinical encounters. The average age of included patients was 67.7 years, with 48.2% of patients being female. Most of the subjects (73.4%) had not had prior shoulder surgery, and 7.6% were current smokers. The most common preoperative diagnosis was shoulder osteoarthritis, seen in 379 patients (49.7%), followed by cuff tear arthropathy (27.8%). Reverse total shoulder arthroplasty was more commonly done compared with aTSA (57.3% vs. 42.7%, respectively). Complete demographic data are presented in Table 1.

**Table 1:** Demographics

Demographic Data	Number	% of Total
Total no. of shoulders	762 shoulders with 1661 clinical encounters	—
No. of patients	721	—
Arthroplasty type		
aTSA	325	42.7%
RSA	437	57.3%
Mean age in years (SD)	67.7 (SD ± 9.1)	
Sex		
Female	395	48.2%
Male	367	51.8%
Mean body mass index (SD)	30.1 (SD ± 6.8)	
Laterality		
Right	462	60.6%
Left	300	39.4%
Prior nonarthroplasty surgery		
Yes	203	26.6%
No	559	73.4%
Comorbidities		
Hypertension	382	50.1%
Heart disease	113	14.8%
Diabetes mellitus	127	16.7%
Current smoker	58	7.6%
Preoperative diagnosis		
Osteoarthritis	379	49.7%
Cuff tear arthropathy	212	27.8%
Inflammatory arthritis	47	6.2%
Fracture sequelae	44	5.8%
Massive rotator cuff tear	34	4.5%
Acute fracture	18	2.4%
Dislocation arthropathy	15	2%
Osteonecrosis	11	1.4%
Tumor	2	0.3%

aTSA = anatomic total shoulder, RSA = reverse total shoulder arthroplasty

**Figure 2:**
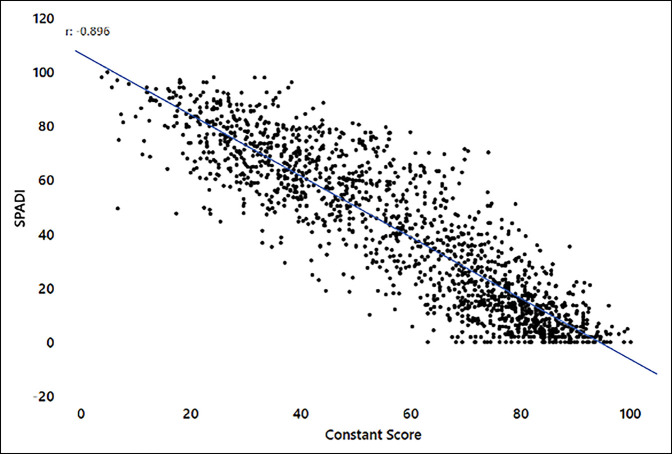
Normalized Constant score (nCS) versus Shoulder Pain and Disability Index (SPADI).

**Figure 3:**
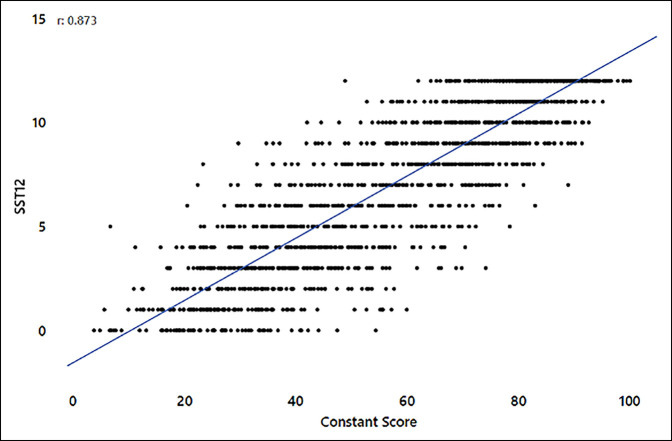
Normalized Constant score (nCS) versus Simple Shoulder Test (SST).

The nCS correlated strongly with each of the PROMs overall: ASES (0.893, *P* < 0.001), SPADI (−0.896, *P* < 0.001), and SST (0.873, *P* < 0.001) (see Figures 1–3). Correlations were similar overall between aTSA and RSA (Supplemental Table 2, http://links.lww.com/JG9/A247). Preoperative correlations between the nCS and the PROMs were on the higher side of moderate correlation with RSA (0.621 to 0.659) and on the lower side of a strong correlation with aTSA (0.704 to 0.705) except for the SST score, which was on the higher side of a moderate correlation (0.608) (Supplemental Table 2, http://links.lww.com/JG9/A247). The 1- and 2-year time points all showed strong correlation of the nCS with PROMs, with the exception of SST in RSA, which showed a moderate correlation that was less than one-hundredth point away from being a strong correlation (Supplemental Table 2, http://links.lww.com/JG9/A247).

**Figurer 1:**
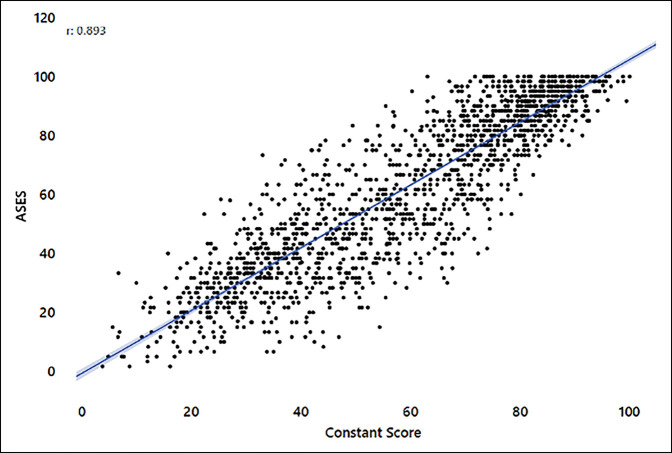
Normalized Constant versus American Shoulder and Elbow Surgeons (ASES) score.

Conversion equations between the nCS and the studied PROMs are detailed in Table 2. The mean differences between the actual and predicted models were less than 0.61 points. The correlation between actual and predicted differences was strong for all equations; however, the within-individual differences between actual and predicted varied widely, demonstrating that these conversion equations are better for population studies compared with individual patient values.

**Table 2:** Conversion Equations and Testing Results Between nCS and Patient-reported Outcome Scores for Shoulder Arthroplasty

Conversion Desired	Conversion Equation	Mean Difference Between Actual and Predicted [95% Confidence Interval]	Correlation Between Actual and Predicted	Within-individual Differences Between Actual and Predicted (range)
ASES → constant	Constant = 18.38 + (ASES × 0.75) − (age × 0.09)	0.37 [−0.57 to 1.32]	0.90	−25.9 to 29.1
SPADI → constant	Constant = 91.91 − (SPADI × 0.72) − (age × 0.06)	−0.34 [−1.32 to 0.63]	0.89	−31.1 to 29.4
SST → constant	Constant = 23.45 + (SST × 5.12) − (age × 0.03)	−0.25 [−1.04 to 0.55]	0.88	−32.9 to 31.9
Constant → SPADI	SPADI = 116.28 − (constant × 1.12) − (age × 0.15)	−0.36 [−1.62 to 0.90]	0.89	−40.7 to 34.9
Constant → ASES	ASES = (constant × −1.12) + (age × −0.19)−12.54	−0.61 [−1.75 to 0.52]	0.90	−32.6 to 33.8
Constant → SST	SST = (constant × 0.15) + (age × 0.02) − 2.54	0.03 [−0.15 to 0.21]	0.88	−5.26 to 5.98

ASES = American Shoulder and Elbow Surgeons, nCS = normalized Constant score, SPADI = Shoulder Pain and Disability Index, SST = Simple Shoulder Test

## Discussion

The results of this study demonstrate that the nCS correlates well with three commonly used PROMs (ASES score, SST, and SPADI). This knowledge is important because it can allow orthopaedic surgeons to decrease their questionnaire burden if desired. Assessment of outcomes after shoulder arthroplasty is derived from numerous individual outcome measures because no single outcome measure is able to reliably evaluate outcomes across diverse patient populations and varying pathology. PROMs use a wide array of parameters that are derived from the patient's judgement on their current condition and are subjective by nature. The Constant score integrates both patient-reported outcomes and a physician's objective findings. Constant scores and PROMs are routinely used in the orthopaedic clinical setting, but there has been minimal correlation between the PROMs and the Constant score. This study demonstrates a high correlation between the nCS and three commonly used PROMs in a large group of shoulder arthroplasty patients.

Correlation among shoulder PROMs has previously been evaluated,^4,8,9,22,23^ but less commonly with more limited studies on shoulder arthroplasty patients.^9,11,17,24,25^ One study evaluating 69 primary RSAs showed good correlation of the ASES score and the Oxford Shoulder Score.^24^ Retzky et al.^9^ showed moderate correlation of preoperative scores and strongly correlated postoperative scores when comparing the single assessment numeric evaluation (SANE) and ASES scores in 33 shoulder arthroplasty patients. One study^17^ demonstrated a high correlation between the ASES score, the SPADI, and the SST in primary shoulder arthroplasty. Unlike previous studies, Michaels et al. evaluated correlations at multiple time points, including preoperatively, and early and late postoperatively. The authors demonstrated that correlations among scores strengthened as time from surgery increased. This echoes our study in that the preoperative correlation between the nCS and PROMs was less compared with the later follow-up time points.

Although the Constant score is a commonly used score in the shoulder literature, it does have some limitations. One review highlighted the need for standardization and measurement precision when reporting the Constant score in the literature given the use of clinician inputted data.^13^ The objective measure of strength has been reported to be measured in a variety of ways, which may affect the final outcome.^12,13,16,26^ In addition, ROM recorded can vary based on the examiner and the method of measurement. One study also showed that the experience of the person performing the measurements markedly affected the final score, but standardization improved the consistency.^27^ For our patients, all measurements were made by experienced research staff in a standardized fashion. Emphasizing concern with the provider-derived data inconsistencies, two articles have evaluated a patient-administered version of these data to be used in the Constant score.^28,29^ In addition to measurement variability, age and sex have also been shown to affect the Constant score in healthy patients.^12,14,30-32^ The nCS was designed to account for how age and sex affect the Constant score so that patients of differing ages can be adequately compared.^14^ Several studies have shown that age and sex affect outcomes in shoulder arthroplasty,^12,33,34^ supporting the use of the nCS for correlations with PROMs in place of the standard Constant score.

The Constant score has been correlated with PROMs in multiple shoulder pathologies,^4,22,23^ but there are few studies looking at correlation of outcome scores with the Constant score in shoulder arthroplasty.^10,11,22,35^ Gilbart et al.^22^ showed that the Constant score correlated moderately (0.69) with the Subjective Shoulder Value in 83 shoulder arthritis patients. Sabesan et al.^10^ only showed moderate correlation of the Constant score with the ASES score (*R* = 0.589) and lesser correlation with the Subjective Shoulder Value score (*R* = 0.361) in 148 reverse shoulder arthroplasties. The Constant score also correlated best with forward elevation and external rotation,^10^ which is not surprising given the physician component of the Constant score. Roy et al.^35^ showed moderate correlation (*R* = 0.78) of the Constant score with the SST in 51 RSA patients with markedly less correlation noted with objective ROM measures. Tuttle et al.^11^ retrospectively analyzed 605 aTSA patients and found that the Constant score has weak-to-moderate correlation with the ASES score (*r* = 0.446), Western Ontario osteoarthritis of the shoulder (WOOS) score (*R* = 0.455), visual analog scale (VAS) for pain score (*R* = 0.302), and the SANE (*R* = 0.180). In addition, this correlation did not change much when using the nCS, and only minimally improved correlation was seen when strength was removed using the modified Constant score.^11^ Our study improves on these previous studies by evaluating a markedly larger cohort of patients, evaluating both aTSA and RSA, focusing on the nCS, and using standardized measurements of strength and ROM in all patients.

The strong correlation of the Constant score with PROMs and the conversion equations have multiple clinical applications. Because different studies in systematic reviews and meta-analyses do not typically use the same outcome measures because of the lack of a benchmark outcome measure, difficulty in analyzing the outcomes often occurs. A lack of uniformity in outcome scores is a common problem seen in the shoulder literature^4,6^ and in the shoulder arthroplasty literature^36,37,38,39^ and has made meaningful comparison across studies difficult. Multiple shoulder arthroplasty meta-analyses and systematic reviews have cited high outcome measure heterogeneity as a notable limitation and also introduce the possibility of inclusion bias.^36,37,39,40^ This study provides a conversion equation between the normalized Constant and multiple commonly used PROMs, which could assist future meta-analyses and systematic reviews in comparing outcomes between studies that used different outcome measures. This information can also allow for multicenter studies that have collected different outcome scores and can help them calculate a common outcome score so that their data can be combined.

Based on our findings, a strong correlation exists between the PROMs and the physician-derived nCS. Scores with objective measurements necessitate more time and input from the physician, making them more arduous to collect and requiring in-person evaluation. Given the strong correlation in shoulder arthroplasty identified by this study between these PROMs with the Constant score, physicians may be able to reduce the patient questionnaire burden and spend less time collecting objective measures without losing meaningful information for future research. Supporting this idea, one study reported that patient satisfaction correlated much better with subjective outcome scores (PROMs) compared with objective measures (strength and ROM) in shoulder arthroplasty patients.^25^

There are several limitations to this study. First, due to the retrospective nature, there may be some selection bias because the patients included were only the patients returning for the routine follow-up. In addition, not every time point could be included for every patient because of lack of data at certain time points. In addition, we acknowledge that PROMs and Constant score measurements are cross-sectional in nature and may not accurately represent a patient's ultimate outcome. However, we guarded against this by recording measurements at multiple time points and eliminating early time points with inferior correlative.^17^ Importantly, the conversion equations were calculated based on a best-fit line; however, individual patient scores varied dramatically (see the within-individual differences between the actual and predicted scores in Table 2). Similarly, the correlation and conversion equations will likely be inaccurate in small cohorts of patients. Therefore, we feel these conversion equations are best used to compare means from pooled population data and are not sufficiently accurate when trying to predict individual patient scores. In addition, the Constant score exhibits less ceiling effects compared with the other outcome measures studied, so correlations in populations with high outcome scores may not be as accurate as presented here.^41^ Finally, PROMs are subjective and may be influenced by patient circumstances on the day of survey completion.

## Conclusion

The nCS shows high correlation with the ASES score, the SPADI, and the SST in primary shoulder arthroplasty patients. This suggests that PROMs may be sufficient for the assessment of shoulder function without the need for clinician input. This knowledge may help to streamline outcome collections and patient survey burden. Finally, the PROM conversion equations generated by our study findings may provide utility in the evaluation of shoulder arthroplasty outcomes across studies in systematic reviews and meta-analyses.
